# The potential mechanisms and regulatory roles of exosomal miRNA in neural repair after spinal cord injury

**DOI:** 10.3389/fncel.2025.1711454

**Published:** 2026-01-20

**Authors:** Yuanhu Shi, Zhilong Li, Yuanxu Pu, Qinghua Wang, Zhiming Cui, Longju Qi, Yuyu Sun

**Affiliations:** 1 School of Medicine and Affiliated Nantong Hospital 3 of Nantong University, Nantong, Jiangsu, China; 2 School of Medicine, Nantong University, Nantong, Jiangsu, China; 3 Laboratory Animal Center, Nantong University, Nantong, Jiangsu, China; 4 Department of Spine Surgery, Nantong City No.1 People's Hospital and The Affiliated Hospital 2 of Nantong University and Research Institute for Spine and Spinal Cord Disease of Nantong University, Nantong, Jiangsu, China; 5 Department of Science and Education in Affiliated Nantong Hospital 3 of Nantong University and Nantong Third People's Hospital, Nantong, Jiangsu, China; 6 Department of Orthopedics in Affiliated Nantong Hospital 3 of Nantong University, Nantong, China

**Keywords:** axonal regeneration, exosomes, microRNA, neuroinflammation, spinal cord injury

## Abstract

Spinal cord injury (SCI) is a devastating disorder of the central nervous system. It is characterized by primary mechanical damage and secondary pathological cascades. These lead to persistent sensory and motor deficits, substantial socioeconomic burdens, and limited therapeutic efficacy. Exosomes are nanoscale vesicles secreted by various cells that serve as key mediators of intercellular communication by delivering bioactive molecules, particularly microRNAs (miRNAs), which regulate gene expression in target cells. This review explores how exosomal miRNAs contribute to neural repair in SCI. These contributions include inhibiting neuroinflammation via pathways such as NF-κB and TLR4; suppressing neuronal apoptosis through PTEN/PI3K/Akt signaling; promoting axonal regeneration via the ERK1/2/STAT3 and NGF/TrkA pathways, enhancing angiogenesis by targeting SPRED1 and integrin α5, and modulating of the immune microenvironment toward M2 polarization, and multifaceted neuroprotection involving alleviating autophagy and endoplasmic reticulum stress. Drawing on recent preclinical studies from 2024–2025, including those utilizing mesenchymal stem cell–derived exosomes loaded with miRNAs such as miR-124-3p, miR-338-5p, and miR-216a-5p, the review highlights promising innovations, such as bioengineered exosomes and biomaterial integrations. Recent preclinical advancements, such as exosome-based therapies that have shown reduced lesion volumes and improved motor function in rodent models, highlight the potential for translation to clinical applications. Ongoing efforts are anticipated to lead to clinical trials in the near future. Despite challenges in standardization, delivery efficiency, immunogenicity, and long-term safety, exosomal miRNA therapy offers a cell-free, multitargeted approach with strong potential for clinical translation in SCI management.

## 1 Part I: Introduction to spinal cord injury

### 1.1 Epidemiology and pathophysiology of spinal cord injury

SCI is a central nervous system injury that is usually caused by traumatic events such as traffic accidents, falls, sports injuries, or violence (Silva et al., 2014). Each year, approximately 10.4–83 per million worldwide suffer from SCI. Moreover, the mortality and morbidity rates are on the rise every year (GBD Spinal Cord Injuries Collaborators., 2023; Yu et al., 2024). The pathological process of SCI can be divided into two stages: primary and secondary injury. Primary injuries are caused by mechanical forces acting directly on the spinal cord, resulting in physical destruction of neurons, axons, and blood vessels (Ahuja et al., 2017). Secondary injury usually occurs within hours to weeks, the pathological process of secondary spinal cord injury is complex and covers a few aspects including hemorrhage and hematomas formation, inflammatory response, ischemia and hypoxia, toxic effects of excitatory amino acids, apoptosis and glial scar formation (Hu et al., 2023; Anjum et al., 2020). After the SCI, blood vessel rupture leads to hemorrhage and hematomas formation, which compresses the nerve tissue and hinders the blood supply, aggravating the local ischemia and hypoxia (Alizadeh et al., 2019); in the inflammatory response, the damage of vascular endothelial cells destroys the blood-spinal cord barrier (BSCB), which leads to the exudation of inflammatory cells and release of inflammatory mediators (Liu et al., 2021), and at the same time, the injured neuronal cells also release cytokines and chemokines, attracting more inflammatory cells, thus forming a vicious circle (Hellenbrand et al., 2021); the energy metabolism of nerve cells is impaired by ischemia and hypoxia, which leads to dysfunction of ion pumps and overloading of intracellular ions, resulting in oedema and rupture of cells (Anjum et al., 2020; Okada, 2016; Ortega et al., 2023; Li et al., 2022b); the release of large amounts of excitatory amino acids excessively activates cell membrane receptors, leading to inward flow of calcium ions and activation of calcium-dependent enzymes to destroy the cellular structure and function (Zrzavy et al., 2021); apoptosis is triggered by a variety of factors, affecting the number of nerve cells surviving; glial scarring has the role of isolating the damaged area, but overgrowth hinders the regeneration and extension of nerve axons (Zrzavy et al., 2021; Orr and Gensel, 2018). In addition, oxidative stress also plays an important role in secondary damage, where excess Reactive Oxygen Species (ROS) can trigger lipid peroxidation, protein denaturation and DNA damage, leading to apoptosis and necrosis (Visavadiya et al., 2016). These secondary reactions further exacerbate neuronal damage and lead to irreversible loss of function (Gris et al., 2008). This complex pathological process ultimately leads to long-term dysfunction and severely reduced quality of life in patients with SCI (Hagen, 2015).

### 1.2 Current treatments and emerging therapies for spinal cord injury

SCI is a devastating condition leading to permanent functional deficits, with current treatments focusing on acute stabilization through surgical decompression, methylprednisolone for polarization, and hemodynamic management to mitigate secondary damage, alongside rehabilitation therapies like physical training and assistive devices to preserve remaining function and prevent complications (Karsy and Hawryluk, 2019). Emerging regenerative approaches include stem cell transplantation, nanomaterials, and gene therapies to promote neural repair (Bartlett et al., 2020; Sugai et al., 2025). Recent advances in exosome-based RNA therapy for SCI highlight its potential as a cell-free therapy (Hwang et al., 2023). From 2024 to 2025, studies showed that exosomes derived from mesenchymal stem cells (MSCs) and neural stem cells can effectively deliver therapeutic RNAs, such as miR-21, miR-124-3p and miR-145-5p, to the site of SCI by crossing the BSCB. In rodent models, these RNAs have been shown to inhibit inflammation through pathways such as NF-κB, reduce neuronal apoptosis, promote axonal regeneration, enhance angiogenesis, and significantly improve motor recovery (Ralph et al., 2024). Innovations include bioengineered exosomes with enhanced miRNA loading (e.g., miR-26a, miR-133b) and integration with biomaterials, such as scaffolds, to optimize delivery and retention (Sousa et al., 2025). For instance, a study demonstrated that bone marrow-derived mesenchymal stem cell exosomes containing miR-338-5p reduced cell apoptosis via the Cnr1/Rap1/Akt axis (Zhang et al., 2021). Another study reported that hypoxia-pretreated bone marrow-derived mesenchymal stem cell exosomes containing miR-216a-5p promoted M2 macrophage polarization (Shao et al., 2024; Liu et al., 2020). Despite challenges such as low yield and standardization, bibliometric analysis indicates a robust trajectory toward clinical translation, with ongoing trials exploring safety and efficacy. To further illustrate the translational potential, recent preclinical studies from 2024–2025 have paved the way for clinical applications. For example, a 2025 study on exosome-based therapy for SCI in rodent models demonstrated nerve regeneration and functional recovery, with plans for human clinical trials announced for 2026. These developments, combined with interdisciplinary efforts in bioengineering, suggest exosomal miRNA therapy could soon enter broader clinical testing.

## 2 Part II: Introduction to exosomes and exosomal miRNAs

### 2.1 Biogenesis and composition of exosomes

Exosomes are nanoscale, membrane-bound vesicles that are actively secreted by nearly all cell types into the extracellular space under physiological or pathological conditions. They typically range in diameter from 30 to 150 nanometers. They act as “messengers” for intercellular communication, stably carrying and transporting various bioactive molecules from source cells within their lipid bilayer membranes. The contents of exosomal vesicles are particularly important and include proteins, lipids, and nucleic acids, especially functional RNAs such as messenger RNA (mRNA), miRNA, and lncRNA (Kalluri and Lebleu, 2020). These exosomal RNAs are not passively loaded, but rather are products of cellular sorting mechanisms. After being delivered to recipient cells, they can directly participate in and regulate gene expression networks, thereby playing a crucial role in remodeling the microenvironment and regulating cellular function.

### 2.2 Advantages of exosomes as delivery vehicles in spinal cord injury treatment

**Figure 1:**
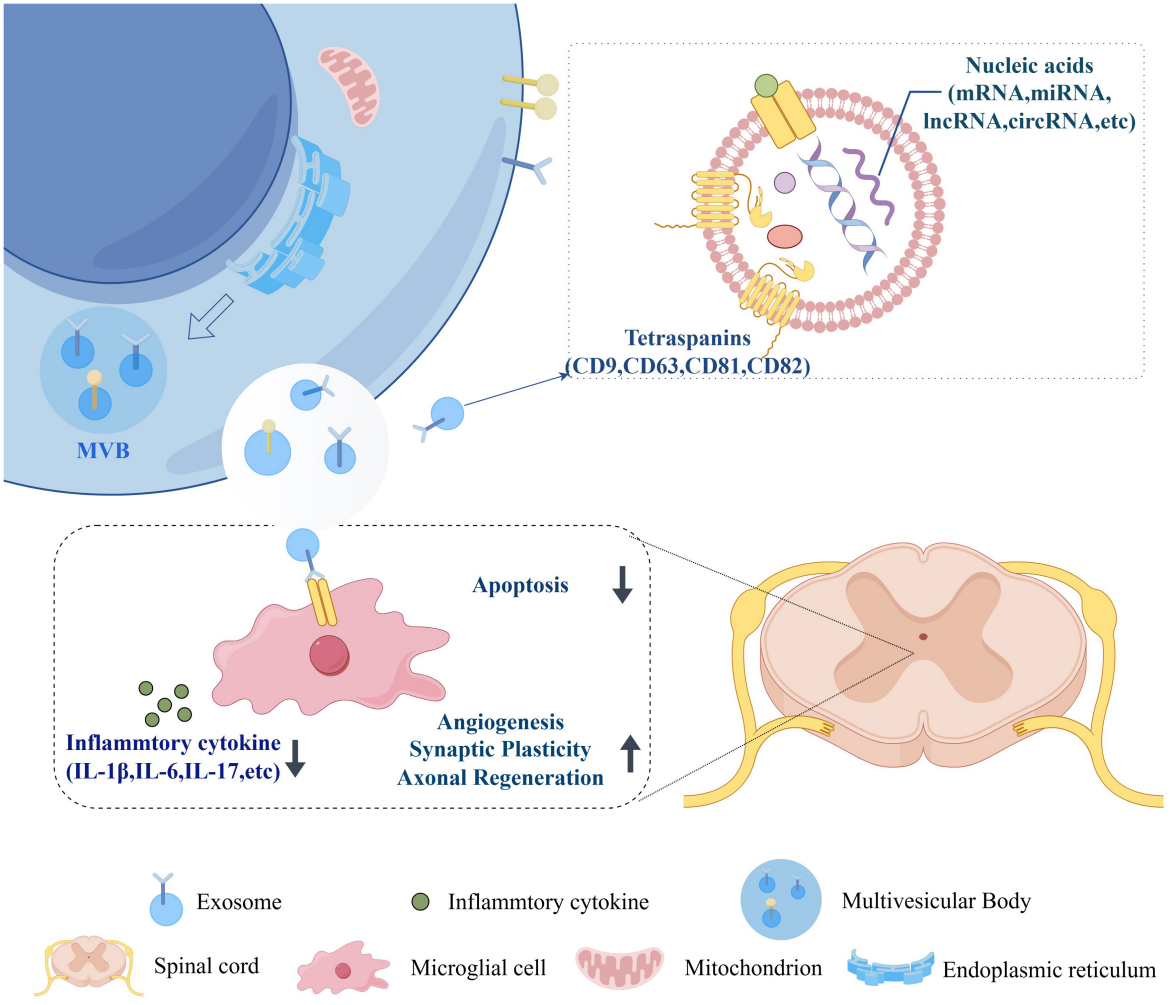
Biogenesis and functional regulation of exosomes in the spinal cord microenvironment.

Exosomes' unique property as natural information carriers gives them unparalleled advantages in the treatment of SCI (Tan et al., 2024; Li et al., 2024). The pathological microenvironment following SCI is extremely complex. RNA molecules, which can regulate multiple key pathways, such as inflammation, apoptosis, angiogenesis, and axonal regeneration, simultaneously, are considered highly promising therapeutic tools (Figure 1) (Gaudet and Popovich, 2014). However, naked RNA molecules are highly unstable *in vivo*. They are prone to rapid degradation by nucleases and struggle to penetrate physiological barriers. This severely limits their clinical application. Exosomes effectively address these challenges. First, their natural membrane structure provides robust protection for the internal RNA, shielding it from degradation. Second, they exhibit excellent biocompatibility and low immunogenicity, ensuring the safety of the delivery process. More critically, they can cross the blood-brain barrier, enabling them to deliver RNA directly to the central nervous system's core injury site (Luan et al., 2017). Therefore, using exosomes as RNA delivery carriers is a strategic “empowerment” that significantly amplifies the therapeutic efficacy of RNA. This opens up a promising path for developing efficient, safe, new strategies for spinal cord injury repair.

### 2.3 Role of exosomal non-coding RNAs with a focus on miRNAs

Exosomes carry a variety of RNA molecules that form a complex regulatory network which participates in the repair process following spinal cord injury. Key non-coding RNA molecules include miRNAs, lncRNAs, circRNAs, and others. LncRNAs and circRNAs can act as “molecular sponges” to absorb miRNAs or participate in other complex regulatory mechanisms, playing a role in neural regeneration and functional remodeling (Wu et al., 2022). However, miRNAs are undoubtedly the most studied and mechanistically well-understood class of molecules in current research (Silvestro and Mazzon, 2022). They precisely “silence” the expression of specific genes at the post-transcriptional level by binding to the mRNA of target genes (Bartel, 2018). This efficiently regulates key pathological processes, such as inflammatory responses, cell apoptosis, and axonal growth (Yang et al., 2022; Pan et al., 2021). Due to significant breakthroughs in the study of miRNAs in spinal cord injury repair and relatively clear mechanisms, this review will focus on miRNAs delivered via exosomes (Table 1). It will systematically elucidate their mechanisms of action and application prospects in spinal cord injury treatment.

**Table 1:** Mechanisms and therapeutic potential of different miRNAs in spinal cord injury.

**miRNAs**	**Donor cells**	**Pathway**	**Function**	**Exosomes administration method**	**References**
miR-9-5p	BMSCs	HDAC5/FGF2	Alleviate apoptosis, inflammation, ER stress	Tail vein injection	He et al., 2022 (PMID: 35316648)
miR-21	MSCs	PTEN/PDCD4	Inhibit neuronal apoptosis	Intravenous injection	Yang et al., 2024 (PMID: 37641874)
miR-21-5p	MSCs or iPSCs	PTEN/PDCD4	Enhance neuronal viability, inhibit neuronal apoptosis	Intravenous injection	Li M. et al., 2025 (PMID: 31217294)
miR-19b	MSCs	PTEN/PDCD4	Enhance neuronal viability, inhibit neuronal apoptosis		Xu et al., 2019a (PMID: 31217294)
miR-23b	BMSCs	TLR4/NF-κB	Relieve inflammatory response		Nie and Jiang, 2021 (PMID: 35109752)
miR-24-3p	BMSCs	MAPK9/JNK/c-Jun/c-Fos	Inhibit neuronal inflammation		Li D. et al., 2025 (PMID: 40274174)
miR-26a	MSCs	PTEN/AKT/mTOR	Promote axonal regeneration	Tail vein injection	Chen et al., 2021 (PMID: 33820561)
miR-26a-5p	BMSCs	EZH2/BDNF/TrkB/CREB	Promote BDNF expression	Intravenous injection	Chen M. et al., 2024 (PMID: 38478142)
miR-27a-3p	UCMSCs	DLL4	Promote angiogenesis	Intranasal administration	Sun et al., 2024b (PMID: 38519036)
miR-29b	BMSCs	PTEN/caspase-3	Inhibit neuronal apoptosis	Intravenous injection	Yu et al., 2019 (PMID: 31819694)
miR-29b-3p	hucMSCs	PTEN/Akt/mTOR	Alleviate neuronal injury, reduce inflammation	Tail vein injection	Xiao et al., 2021 (PMID: 34385115)
miR-34a-5p	NSCs	HDAC6	Promote neural regeneration, activate autophagy	Local delivery	Qin et al., 2024 (PMID: 38059122)
miR-92a-3p	hucMSCs	PTEN/AKT/mTOR	Inhibit neuronal apoptosis		He et al., 2020 (PMID: 32297644)
miR-124-3p	BMSCs	PI3K/AKT/NF-κB	Suppress neurotoxic microglia and astrocytes	Tail vein injection	Jiang et al., 2020 (PMID: 32711535)
miR-125a	BMSCs	IRF5	Promote M2 macrophage polarization	Intravenous injection	Chang et al., 2021 (PMID: 33609602)
miR-125a-3p	MSCs		Deactivate NET formation	Intravenous injection	Chen H. et al., 2024 (PMID: 38397083)
miR-126	MSCs	SPRED1/PIK3R2	Promote angiogenesis and neurogenesis, attenuate apoptosis	Tail vein injection	Huang et al., 2020 (PMID: 31704348)
miR-133b	MSCs	ERK1/2/STAT3	Promote axonal regeneration, improve functional recovery	Tail vein injection	Li et al., 2018 (PMID: 30524227)
miR-137	BMSCs	TLR4/NF-κB	Improve locomotor capacity, reduce tissue injury	Intravenous injection	Zhang et al., 2023 (PMID: 36596504)
miR-145-5p	MSCs	TLR4/NF-κB	Reduce inflammation	Intravenous injection	Jiang and Zhang, 2021 (PMID: 33945431)
miR-146a-5p	MSCs	IRAK1/TRAF6/NF-κB	Promoting M2 Macrophage Polarization	Intravenous injection	Liang et al., 2024 (PMID: 38488560)
miR-151-3p	Microglia	p53/p21/CDK1	Attenuate neuronal apoptosis, enhance healing		Li et al., 2022a (PMID: 35127706)
miR-181c	BMSCs	TNF-α/IL-1β	Inhibit inflammation and apoptosis	Tail vein injection	Zhang et al., 2021 (PMID: 33548000)
miR-199a-3p	Umbilical MSCs	NGF/TrkA	Facilitate functional recovery	Tail vein injection	Wang et al., 2021 (PMID: 33579361)
miR-210-5p	Pericytes	JAK1/STAT3	Improve mitochondrial function, inhibit lipid peroxidation		Gao et al., 2023 (PMID: 38012616)
miR-216a-5p	MSCs (hypoxic)	TLR4/NF-κB	Shift microglial M1/M2 polarization, repair SCI	Tail vein injection	Liu et al., 2020 (PMID: 32019561)
miR-219a-2-3p	NSCs	YY1/NF-κB	Neuroprotective effects, inhibit inflammation	Tail vein injection	Ma et al., 2019 (PMID: 31848325)
miR-329-3p	MSCs	IGF1R	Improve SCI recovery		Liu J. et al., 2022 (PMID: 34623606)
miR-338-5p	MSCs	Cnr1/Rap1/Akt	Neuroprotective, reduce apoptosis, promote survival	Tail vein injection	Zhang et al., 2021 (PMID: 34302891)
miR-374-5p	NSCs	STK-4/autophagy	Inhibit neuronal apoptosis		Zhang and Han, 2022b (PMID: 36046998)
miR-381	MSCs	BRD4/WNT5A	Inhibit neuronal apoptosis	Intravenous injection	Jia et al., 2021 (PMID: 34024119)
miR-429	Plasma	PTEN/PI3K/Akt	Inhibit neuronal apoptosis		Huang et al., 2022 (PMID: 35242851)
miR-431-3p	BMSCs	RGMA	Promote axon regeneration		Sun et al., 2024a (PMID: 38227255)
miR-455-5p	BMSCs	TIAM1	Protect against ischemia reperfusion injury, promote autophagy	Tail vein injection	Liu B. et al., 2022 (PMID: 34823099)
miR-494	BMSCs	PTEN/PDCD4	Promote neurofilament regeneration, behavioral recovery	Tail vein injection	Wang et al., 2021 (PMID: 34635862)
miR-544	BMSCs	IRF5	Promote functional recovery, attenuate inflammation	Tail vein injection	Li et al., 2020 (PMID: 32141339)

## 3 Part III: Functions of exosomal miRNA in the treatment of spinal cord injury

### 3.1 Inhibition of neuroinflammatory responses

Neuroinflammation is the primary mechanism that drives secondary pathological cascade reactions following SCI. Following injury, the release of numerous pro-inflammatory cytokines, such as IL-1β and TNF-α, activates signaling pathways, including NF-κB and TLR4 (Ransohoff, 2016; Wang et al., 2015). This exacerbates neuronal damage and deteriorates the local microenvironment. Exosome-derived miRNAs can target these pathways precisely or directly regulate pro-inflammatory transcription factors, such as IRF5 (Gong et al., 2024). This effectively inhibits excessive activation of microglia and macrophages, mitigating the inflammatory response (Yuan et al., 2023). The therapeutic value of exosomes lies in breaking the vicious cycle of inflammation, reducing secondary neural damage, and creating favorable conditions for tissue repair and functional recovery (Nakazaki et al., 2021). For instance, Jiang et al. showed that Neural stem cells (NSC) exosome-derived miR-124-3p inhibits the PI3K/AKT/NF-κB pathway by targeting MYH9. This significantly reduces pro-inflammatory factor levels and improves motor function in SCI rat models (Jiang et al., 2020). From a systems biology perspective, miRNAs like miR-124-3p interact with multiple pathways and cell types in the SCI microenvironment, forming competing endogenous RNA (ceRNA) networks that modulate inflammation across microglia, astrocytes, and macrophages. Integrated multi-omics profiling in rat models has revealed miRNA-guided regulatory networks post-SCI, where miRNAs orchestrate gene expression hubs involving inflammation, apoptosis, and regeneration, highlighting their role as central nodes in dynamic cellular interactions (Klassen et al., 2025). Similarly, Liu et al. (2020) found that hypoxia-pretreated MSC exosomes regulate the TLR4/NF-κB pathway via miR-216a-5p, inducing microglia to polarize from the pro-inflammatory M1 type to the anti-inflammatory M2 type. Recent evidence indicates that hypoxia-preconditioned bone marrow mesenchymal stem cell (BMSC)-derived exosomes enriched with miR-146a-5p can promote M2 polarization of macrophages by modulating the IRAK1-TRAF6-NF-κB signaling axis. This alleviates neuroinflammation and improves the post-injury microenvironment (Liang et al., 2024). Together, these studies underscore the significant potential of exosome miRNA therapy in precisely regulating the immune microenvironment.

### 3.2 Inhibition of neuronal apoptosis

Neuronal apoptosis is a key factor in permanent functional loss following SCI, involving mitochondrial dysfunction, oxidative stress, and abnormal upregulation of pro-apoptotic genes, such as PTEN and Bax (Liu et al., 2019). Exosomal miRNAs can reduce neuronal apoptosis by targeting and inhibiting these pro-apoptotic genes or by activating key cell survival pathways (Feng et al., 2021), such as the PI3K/AKT pathway (Huang et al., 2022; Xiao et al., 2022). Maintaining the survival of neural networks is essential for axonal regeneration and functional reconstruction; therefore, inhibiting apoptosis is crucial for extending the therapeutic window (Huang et al., 2020). Zhang et al. (2021) demonstrated that BMSC-derived miR-338-5p regulates the Cnr1/Rap1/Akt axis by upregulating the anti-apoptotic protein Bcl-2 and downregulating Bax, significantly reducing neuronal apoptosis in SCI rats. Systems biology analyses indicate that miRNAs such as miR-338-5p engage in multifaceted interactions, regulating apoptosis through interconnected pathways involving neurons, endothelial cells, and immune cells, as evidenced by whole transcriptome sequencing revealing miRNA-mRNA networks that balance pro- and anti-apoptotic signals in the SCI niche (Klassen et al., 2025). Additionally, Huang et al. found that plasma exosomal miR-429 can reduce neuronal loss during the acute SCI phase by inhibiting the PTEN/PI3K/Akt pathway (Huang et al., 2022). Furthermore, endothelial progenitor cell-derived exosomes loaded with miR-210 were shown to decrease the Bax/Bcl-2 ratio and cleaved caspase-3 levels. This led to improved BBB scores from days 7 to 28 post-injury (Wang et al., 2024). These findings suggest that exosomal miRNA therapies targeting apoptosis offer significant neuroprotective advantages.

### 3.3 Promoting axonal regeneration

Axonal regeneration forms the structural basis for the recovery of motor and sensory function following SCI. However, the glial scar formed after injury and the accumulation of inhibitory molecules (Hesp et al., 2015), such as Repulsive Guidance Molecule A (RGMA) (Nakagawa et al., 2019), in the microenvironment, are the primary obstacles to axonal growth. Exosomal miRNAs can effectively promote axonal extension and reconstruction of functional neural circuits by regulating signaling pathways critical for axonal growth, such as the PTEN/AKT/mTOR and NGF/TrkA pathways (Li et al., 2018). Wang et al. discovered that human umbilical cord MSC exosomal miR-145-5p can significantly promote axonal growth in SCI rat models by activating the NGF/TrkA pathway (Wang et al., 2021). Similarly, (Li et al. 2018) confirmed that miR-133b, when delivered by modified MSC exosomes, activates the ERK1/2/STAT3 pathway, thereby stimulating nerve fiber regeneration. Incorporating a systems biology lens, miR-133b and similar miRNAs interact with diverse cell types (e.g., neurons and oligodendrocytes) via regulatory networks, as shown in multi-omics studies where miRNA hubs coordinate axonal guidance, myelin repair, and extracellular matrix remodeling in the SCI microenvironment (Klassen et al., 2025). Additionally, a study demonstrated that epidermal growth factor receptor (EGFR)-positive neural stem cell-derived exosomes carrying miR-34a-5p promote axonal growth and enhance functional recovery after spinal cord injury by silencing histone deacetylase 6 (HDAC6). This finding confirms the critical role of neural exosomes in axonal regeneration (Qin et al., 2024). While these studies highlight the immense potential of exosomal miRNAs in overcoming regenerative barriers, addressing the complexity and persistence of glial scar formation remains a key challenge in optimizing therapeutic outcomes.

### 3.4 Promoting angiogenesis

Vascular reconstruction in the injured area is crucial for improving local ischemia and hypoxia, supporting neuronal survival, and removing metabolic waste. It is also a prerequisite for optimizing the neuro-repair microenvironment (Xin et al., 2013). Exosomal miRNAs can promote endothelial cell proliferation, migration, and tubular structure formation by targeting and regulating genes related to angiogenesis (e.g., integrin α5 and SPRED1) (Anderson et al., 2016; Zhang et al., 2015). Umezu et al. (2013) demonstrated that miR-92a, delivered by K562 cell exosomes, significantly increased neovascular density in a SCI model by targeting integrin α5. Huang et al. (2020) found that miR-126 in modified MSC exosomes increases the expression of angiogenesis markers [e.g., vascular endothelial growth factor (VEGF)] by regulating the SPRED1/PIK3R2 pathway, thereby promoting functional recovery. In systems biology terms, miR-126 forms part of integrated networks linking endothelial cells with neurons and immune cells, modulating angiogenesis through feedback loops identified in omics-based models of the SCI vascular niche (Klassen et al., 2025; Zhang et al., 2023). Furthermore, it was found that exosomes secreted by human urine-derived stem cells are enriched in miR-216a-5p. This microRNA targets PTEN and activates the AKT signaling pathway, promoting angiogenesis and enhancing cell survival. This provides new mechanistic insight into exosome-mediated vascular reconstruction (Zhang et al., 2020). These findings underscore the pivotal role of exosomal miRNAs in angiogenesis.

### 3.5 Regulating the immune microenvironment

An imbalance in the immune microenvironment after SCI, particularly the excessive activation of M1-type pro-inflammatory macrophages/microglia, exacerbates inflammatory damage and disrupts the integrity of the BSCB (Milich et al., 2019). Exosomal miRNAs can regulate key signaling pathways to induce polarization of macrophages/microglia toward an M2-type reparative phenotype (Liu et al., 2020). This protects the structural integrity of the BSCB and reshapes the immune microenvironment from “damaging” to “reparative” (Noble and Wrathall, 1989). Qing et al. demonstrated that miRNAs in BMSC exosomes (e.g., miR-125a) promote M2 polarization by regulating the PI3K/AKT/NF-κB pathways (Chang et al., 2021). Additionally, Gao et al. (2023) found that pericardial cell exosomal miR-210-5p can protect the BSCB's integrity through the JAK1/STAT3 pathway. These findings underscore the pivotal role of exosomal miRNAs in immune regulation.

### 3.6 Multifunctional neuroprotective effects

In addition to their specific functions, certain exosomal miRNAs have broad-spectrum neuroprotective effects. These effects are achieved through multiple mechanisms, including regulating cellular autophagy, alleviating endoplasmic reticulum (ER) stress, and promoting neuronal differentiation (Zhang and Han, 2022b; Li R. Y. et al., 2023; He et al., 2022). These multifaceted, multitargeted effects provide a foundation for improving the overall neural microenvironment following SCI and supporting long-term functional recovery (Chang et al., 2024). For instance, Ke et al. discovered that neural stem cell exosomes enhanced by IGF-1 exert neuroprotective effects through the miR-219a-2-3p/YY1 axis (Ma et al., 2019). Xu et al. (2019b) demonstrated that miR-92b-3p in astrocyte exosomes provides early protection during the acute phase of SCI by alleviating endoplasmic reticulum stress. Additionally, a study published in the journal Pain revealed that exosomal miRNAs modulate neuropathic pain following SCI by affecting neuronal hyper-excitability pathways. This finding expands our understanding of exosome-mediated neuroregulation (Picco et al., 2025). Together, these studies reveal the broad neuroprotective potential of exosomal miRNAs and offer new insights for comprehensive SCI treatment. However, their long-term efficacy and reproducibility in human clinical trials must be validated.

## 4 Part IV: Conclusion

**Figure 2:**
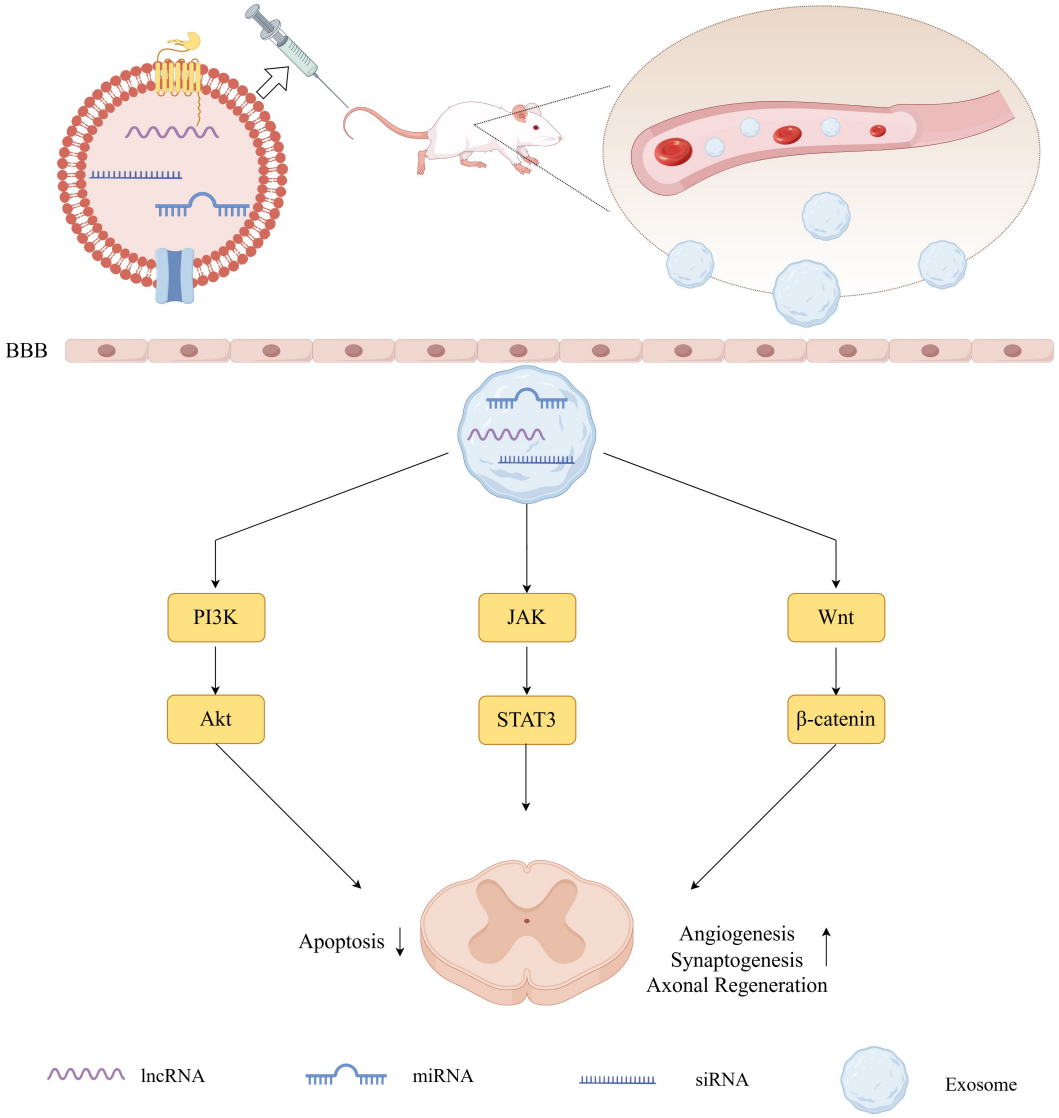
The main pathways of the roles of miRNAs in spinal cord injury.

In summary, SCI remains a significant clinical challenge, involving complex pathophysiological processes that result in permanent neurological impairment. Exosomal RNAs, particularly miRNAs, may emerge as transformative therapeutics, modulating intercellular communication, and orchestrating multifaceted repair processes. As detailed in this review, exosomal miRNAs exert profound effects on SCI pathophysiology by attenuating neuroinflammation through pathways such as NF-κB and TLR4, inhibiting neuronal apoptosis via PTEN/PI3K/Akt signaling, promoting axonal regeneration and angiogenesis, and reshaping the immune microenvironment toward a reparative phenotype (Figure 2) (Zha, 2025). Recent advancements highlight the potential of exosomes derived from MSCs and neural stem cells. Engineered variants of these exosomes can enhance miRNA delivery, leading to better outcomes in preclinical models, such as reduced lesion volumes and improved motor function (Wang et al., 2025; Lin et al., 2025). However, there are still some challenges to overcome before this can be translated into clinical practice, such as standardizing exosome production, optimizing delivery strategies, and mitigating immunogenicity. This requires rigorous GMP-compliant protocols and long-term safety evaluations (Chen et al., 2025; Ma Y. et al., 2025).

From a broader perspective, based on the comprehensive evidence presented in this review, exosomal miRNA therapy represents a paradigm shift in SCI treatment, offering a non-invasive, multitargeted strategy that could bridge the gap between preclinical promise and clinical reality. Its ability to integrate with emerging technologies, such as bioengineered scaffolds and CRISPR-based enhancements, underscores the need for accelerated interdisciplinary collaboration to refine scalability and personalize interventions, which may gradually contribute to more effective and patient-centered regenerative strategies in the future.

## 5 Part V: Challenges and future perspectives for clinical translation

### 5.1 Current challenges in exosomal miRNA therapy for SCI

Exosomal miRNA therapy shows significant potential for repairing SCI, but its clinical translation still faces multiple technical and biological challenges. First, there are prominent standardization issues in exosome preparation, including separation purity, miRNA loading efficiency, and batch-to-batch variability, which may lead to inconsistent therapeutic effects (Li et al., 2024; Zha, 2025). Second, delivery efficiency is limited. Intravenous administration often results in rapid hepatic clearance and insufficient BSCB penetration. Local injection enhances targeting but increases the risk of invasiveness and infection. Dose optimization is also challenging because excessive doses may trigger immune responses or off-target effects that affect gene regulation in non-neural tissues (Singh et al., 2024). In addition, safety considerations are critical as well, including the immunogenicity of donor-derived exosome sources, which may induce inflammation or rejection reactions (Alvi et al., 2024). Long-term risks, such as tumorigenesis, must be monitored vigilantly, as miRNA regulation may interfere with cell proliferation pathways (Wei et al., 2025). Strategies to mitigate these risks encompass the use of autologous or hypoimmunogenic exosomes, surface modification with targeting moieties to enhance specificity, and comprehensive post-treatment monitoring via advanced imaging and biomarker assays to detect any adverse events early (Ma Y. et al., 2025; Wu et al., 2025). Additionally, preclinical studies often neglect gender differences and long-term outcome assessments, limiting their applicability to humans (Shang et al., 2025). Notably, studies have revealed gender differences in the response of extracellular vesicles following chronic spinal cord injury. These differences correlate with neuroinflammation and neurodegenerative changes in the aging brain. Consequently, overlooking gender variations may limit the universality of therapeutic outcomes (Li Y. et al., 2023). To overcome these obstacles, GMP-compliant protocols and biomarker monitoring systems must be developed to ensure the therapy's reliability and safety (Ma Y. et al., 2025).

### 5.2 Future directions and innovations in exosomal miRNA-based treatments

Looking ahead, exosomal miRNA therapy shows promise for making breakthroughs in SCI treatment through multidisciplinary integration. One emerging trend is the optimization of engineered exosomes through hormone pretreatment or induction with specific factors to enhance the anti-inflammatory and regenerative efficacy of miRNAs. Melatonin-pretreated plasma exosomes, for instance, have been shown to significantly enhance anti-inflammatory function by delivering miR-138-5p, thereby inhibiting microglial activation and promoting spinal cord repair. This demonstrates their potential to reduce lesion volume and improve motor function in animal models (Chen H. et al., 2024). Similarly, IL-4-pretreated human umbilical cord mesenchymal stem cell-derived exosomes loaded with miR-125a promote M2 macrophage polarization and downregulate IRF5 expression. Recent studies have reported their effective mitigation of inflammatory cascades in SCI models (Li M. et al., 2025; Liao et al., 2025). Combination strategies are also emerging. For instance, the co-administration of exosomes with biomaterials, such as hydrogel scaffolds, or CRISPR tools can promote synergistic repair mechanisms and sustained release. Studies using GelMA/HA-NB hydrogels for local exosome delivery demonstrated sustained exosome release and nearly doubled functional recovery compared to free exosome administration. These results highlight the potential of biomaterial-integrated delivery strategies for SCI (Cheng et al., 2021). Ultrasound-targeted microbubble destruction enhances the targeted delivery of platelet-rich plasma-derived exosomes to injured peripheral nerves, promoting Schwann cell proliferation and nerve regeneration in rat models (Yi et al., 2023). Recent advances include NT3-loaded, exosome-degradable, conductive scaffolds, which a 2025 study demonstrated to have advantages in promoting axonal regeneration and functional recovery, offering a novel SCI repair platform (Ma L. et al., 2025). Additionally, scalable, off-the-shelf exosome-mimicking nanoparticles will accelerate the translation of research from the laboratory to the clinic, and a surge in clinical trials is expected by 2030 (Kim et al., 2024; Ke et al., 2024). Future research should prioritize the development of personalized exosomal miRNA therapies tailored to individual patient profiles, such as miRNA expression patterns post-injury. Combination therapies integrating exosomal miRNAs with stem cell transplants, biomaterials, or pharmacological agents could synergistically enhance repair mechanisms (Guo et al., 2026). Fostering interdisciplinary collaboration among neuroscientists, bioengineers, and clinicians will be essential to accelerate clinical translation, potentially through multi-center trials and shared databases for real-time data integration.

## References

[B1] Ahuja C. S. Nori S. Tetreault L. Wilson J. Kwon B. Harrop J. . (2017). Traumatic spinal cord injury-repair and regeneration. Neurosurgery 80, S9–S22. doi: 10.1093/neuros/nyw080 28350947

[B2] Alizadeh A. Dyck S. M. Karimi-Abdolrezaee S. (2019). Traumatic spinal cord injury: an overview of pathophysiology, models and acute injury mechanisms. Front. Neurol. 10:282. doi: 10.3389/fneur.2019.00282 30967837 PMC6439316

[B3] Alvi M. A. Pedro K. M. Quddusi A. I. Fehlings M. G. (2024). Advances and challenges in spinal cord injury treatments. J. Clin. Med. 13:4101. doi: 10.3390/jcm13144101 39064141 PMC11278467

[B4] Anderson J. D. Johansson H. J. Graham C. S. Vesterlund M. Pham M. T. Bramlett C. S. . (2016). Comprehensive proteomic analysis of mesenchymal stem cell exosomes reveals modulation of angiogenesis via nuclear factor-KappaB signaling. Stem Cells 34, 601–613. doi: 10.1002/stem.2298 26782178 PMC5785927

[B5] Anjum A. Yazid M. D. Fauzi Daud M. Idris J. Ng A. M. H. Selvi Naicker A. . (2020). Spinal cord injury: pathophysiology, multimolecular interactions, and underlying recovery mechanisms. Int. J. Mol. Sci. 21:7533. doi: 10.3390/ijms21207533 33066029 PMC7589539

[B6] Bartel D. P. (2018). Metazoan MicroRNAs. Cell 173, 20–51. doi: 10.1016/j.cell.2018.03.006 29570994 PMC6091663

[B7] Bartlett R. D. Burley S. Ip M. Phillips J. B. Choi D. (2020). Cell therapies for spinal cord injury: trends and challenges of current clinical trials. Neurosurgery 87, E456–e72. doi: 10.1093/neuros/nyaa149 32497197

[B8] Chang C. Weiping L. Jibing C. (2024). Exosomal MiRNA therapy for central nervous system injury diseases. Cell Mol. Neurobiol. 45:3. doi: 10.1007/s10571-024-01522-0 39652146 PMC11628439

[B9] Chang Q. Hao Y. Wang Y. Zhou Y. Zhuo H. Zhao G. (2021). Bone marrow mesenchymal stem cell-derived exosomal microRNA-125a promotes M2 macrophage polarization in spinal cord injury by downregulating IRF5. Brain Res. Bull. 170, 199–210. doi: 10.1016/j.brainresbull.2021.02.015 33609602

[B10] Chen H. Sun H. Yang Y. Wang P. Chen X. Yin J. . (2024). Engineered melatonin-pretreated plasma exosomes repair traumatic spinal cord injury by regulating miR-138-5p/SOX4 axis mediated microglia polarization. J. Orthop. Transl. 49, 230–245. doi: 10.1016/j.jot.2024.09.007 39512441 PMC11541837

[B11] Chen M. Lin Y. Guo W. Chen L. (2024). BMSC-derived exosomes carrying miR-26a-5p ameliorate spinal cord injury via negatively regulating EZH2 and activating the BDNF-TrkB-CREB signaling. Mol. Neurobiol. 61, 8156–8174. doi: 10.1007/s12035-024-04082-y 38478142

[B12] Chen R. Zheng J. Hao J. Yang Y. Xu S. Zhang F. . (2025). Exosome-loaded bioscaffolds for spinal cord injuries: a review. Stem Cells Int. 2025:8841129. doi: 10.1155/sci/8841129 40771301 PMC12328054

[B13] Chen Y. Tian Z. He L. Liu C. Wang N. Rong L. . (2021). Exosomes derived from miR-26a-modified MSCs promote axonal regeneration via the PTEN/AKT/mTOR pathway following spinal cord injury. Stem. Cell Res. Ther. 12:224. doi: 10.1186/s13287-021-02282-0 33820561 PMC8022427

[B14] Cheng J. Chen Z. Liu C. Zhong M. Wang S. Sun Y. . (2021). Bone mesenchymal stem cell-derived exosome-loaded injectable hydrogel for minimally invasive treatment of spinal cord injury. Nanomedicine 16, 1567–1579. doi: 10.2217/nnm-2021-0025 34189939

[B15] Feng J. Zhang Y. Zhu Z. Gu C. Waqas A. Chen L. (2021). Emerging exosomes and exosomal MiRNAs in spinal cord injury. Front. Cell Dev. Biol. 9:703989. doi: 10.3389/fcell.2021.703989 34307384 PMC8299525

[B16] Gao P. Yi J. Chen W. Gu J. Miao S. Wang X. . (2023). Pericyte-derived exosomal miR-210 improves mitochondrial function and inhibits lipid peroxidation in vascular endothelial cells after traumatic spinal cord injury by activating JAK1/STAT3 signaling pathway. J. Nanobiotechnol. 21:452. doi: 10.1186/s12951-023-02110-y 38012616 PMC10680350

[B17] Gaudet A. D. Popovich P. G. (2014). Extracellular matrix regulation of inflammation in the healthy and injured spinal cord. Exp. Neurol. 258, 24–34. doi: 10.1016/j.expneurol.2013.11.020 25017885 PMC4099942

[B18] GBD Spinal Cord Injuries Collaborators. (2023). Global, regional, and national burden of spinal cord injury, 1990-2019: a systematic analysis for the Global Burden of Disease Study 2019. Lancet Neurol. 22, 1026–1047. doi: 10.1016/S1474-4422(23)00287-9 37863591 PMC10584692

[B19] Gong Z. T. Xiong Y. Y. Ning Y. Tang R. J. Xu J. Y. Jiang W. Y. . (2024). Nicorandil-pretreated mesenchymal stem cell-derived exosomes facilitate cardiac repair after myocardial infarction via promoting macrophage M2 polarization by targeting miR-125a-5p/TRAF6/IRF5 signaling pathway. Int. J. Nanomed. 19, 2005–2024. doi: 10.2147/IJN.S441307 38469055 PMC10926597

[B20] Gris D. Hamilton E. F. Weaver L. C. (2008). The systemic inflammatory response after spinal cord injury damages lungs and kidneys. Exp. Neurol. 211, 259–270. doi: 10.1016/j.expneurol.2008.01.033 18384773

[B21] Guo S. Wang Y. Zhao H. Fu H. Lu Y. (2026). Emerging frontiers in microRNA technology: innovations driving precision medicine. Biomaterials 326:123716. doi: 10.1016/j.biomaterials.2025.123716 40974750

[B22] Hagen E. M. (2015). Acute complications of spinal cord injuries. World J. Orthop. 6, 17–23. doi: 10.5312/wjo.v6.i1.17 25621207 PMC4303786

[B23] He S. Wang Z. Li Y. Dong J. Xiang D. Ren L. . (2020). MicroRNA-92a-3p enhances functional recovery and suppresses apoptosis after spinal cord injury via targeting phosphatase and tensin homolog. Biosci. Rep. 40:BSR20192743. doi: 10.1042/BSR20192743 32297644 PMC7199448

[B24] He X. Zhang J. Guo Y. Yang X. Huang Y. Hao D. (2022). Exosomal miR-9-5p derived from BMSCs alleviates apoptosis, inflammation and endoplasmic reticulum stress in spinal cord injury by regulating the HDAC5/FGF2 axis. Mol. Immunol. 145, 97–108. doi: 10.1016/j.molimm.2022.03.007 35316648

[B25] Hellenbrand D. J. Quinn C. M. Piper Z. J. Morehouse C. N. Fixel J. A. Hanna A. S. (2021). Inflammation after spinal cord injury: a review of the critical timeline of signaling cues and cellular infiltration. J. Neuroinflammation 18:284. doi: 10.1186/s12974-021-02337-2 34876174 PMC8653609

[B26] Hesp Z. C. Goldstein E. Z. Miranda C. J. Kaspar B. K. McTigue D. M. (2015). Chronic oligodendrogenesis and remyelination after spinal cord injury in mice and rats. J. Neurosci. 35, 1274–1290. doi: 10.1523/JNEUROSCI.2568-14.2015 25609641 PMC4300327

[B27] Hu X. Xu W. Ren Y. Wang Z. He X. Huang R. . (2023). Spinal cord injury: molecular mechanisms and therapeutic interventions. Sig. Transduct Target Ther. 8:245. doi: 10.1038/s41392-023-01477-6 37357239 PMC10291001

[B28] Huang J. Wu C. Xu G. Sun Y. Gui C. Fu J. . (2022). The decreased expression of miR-429 in plasma exosomes after spinal cord injury inhibits neuronal apoptosis by mediating the PTEN/PI3K/Akt pathway. Ann. Transl. Med. 10:6. doi: 10.21037/atm-21-5561 35242851 PMC8825548

[B29] Huang J. H. Xu Y. Yin X. M. Lin F. Y. (2020). Exosomes derived from miR-126-modified MSCs promote angiogenesis and neurogenesis and attenuate apoptosis after spinal cord injury in rats. Neuroscience 424, 133–145. doi: 10.1016/j.neuroscience.2019.10.043 31704348

[B30] Hwang J. Jang S. Kim C. Lee S. Jeong H. S. (2023). Role of stem cell-derived exosomes and microRNAs in spinal cord injury. Int. J. Mol. Sci. 24:13849. doi: 10.3390/ijms241813849 37762150 PMC10530823

[B31] Jia X. Huang G. Wang S. Long M. Tang X. Feng D. . (2021). Extracellular vesicles derived from mesenchymal stem cells containing microRNA-381 protect against spinal cord injury in a rat model via the BRD4/WNT5A axis. Bone Joint Res. 10, 328–339. doi: 10.1302/2046-3758.105.BJR-2020-0020.R1 34024119 PMC8160032

[B32] Jiang D. Gong F. Ge X. Lv C. Huang C. Feng S. . (2020). Neuron-derived exosomes-transmitted miR-124-3p protect traumatically injured spinal cord by suppressing the activation of neurotoxic microglia and astrocytes. J. Nanobiotechnol. 18:105. doi: 10.1186/s12951-020-00665-8 32711535 PMC7382861

[B33] Jiang Z. Zhang J. (2021). Mesenchymal stem cell-derived exosomes containing miR-145-5p reduce inflammation in spinal cord injury by regulating the TLR4/NF-κB signaling pathway. Cell Cycle. 20, 993–1009. doi: 10.1080/15384101.2021.1919825 33945431 PMC8172161

[B34] Kalluri R. Lebleu V. S. (2020). The biology, function, and biomedical applications of exosomes. Science 367:eaau6977. doi: 10.1126/science.aau6977 32029601 PMC7717626

[B35] Karsy M. Hawryluk G. (2019). Modern medical management of spinal cord injury. Curr. Neurol. Neurosci. Rep. 19:65. doi: 10.1007/s11910-019-0984-1 31363857

[B36] Ke L. Cao Y. Lu Z. Hallajzadeh J. (2024). Advances in different adult stem cell-derived exosomal non-coding RNAs for the treatment of neurological disorders: a narrative review. Front. Cell Dev. Biol. 12:1459246. doi: 10.3389/fcell.2024.1459246 39450275 PMC11500198

[B37] Kim H. I. Park J. Zhu Y. Wang X. Han Y. Zhang D. (2024). Recent advances in extracellular vesicles for therapeutic cargo delivery. Exp. Mol. Med. 56, 836–849. doi: 10.1038/s12276-024-01201-6 38556545 PMC11059217

[B38] Klassen R. A. Chytilova S. Arzhanov I. Zucha D. Rohlova E. Androvic P. . (2025). Integrated multi-omics profiling uncovers miRNA-guided regulatory networks after spinal cord injury in rats. Mol. Ther. Nucleic Acids 36:102746. doi: 10.1016/j.omtn.2025.102746 41245488 PMC12617769

[B39] Li C. Li X. Zhao B. Wang C. (2020). Exosomes derived from miR-544-modified mesenchymal stem cells promote recovery after spinal cord injury. Arch. Physiol. Biochem. 126, 369–375. doi: 10.1080/13813455.2019.1691601 32141339

[B40] Li C. Qin T. Liu Y. Wen H. Zhao J. Luo Z. . (2022a). Microglia-derived exosomal microRNA-151-3p enhances functional healing after spinal cord injury by attenuating neuronal apoptosis via regulating the p53/p21/CDK1 signaling pathway. Front. Cell Dev. Biol. 9:783017. doi: 10.3389/fcell.2021.783017 35127706 PMC8811263

[B41] Li C. Wu Z. Zhou L. Shao J. Hu X. Xu W. . (2022b). Temporal and spatial cellular and molecular pathological alterations with single-cell resolution in the adult spinal cord after injury. Sig. Transduct Target Ther. 7:65. doi: 10.1038/s41392-022-00885-4 35232960 PMC8888618

[B42] Li D. Xie X. Ou Y. Sun P. Lin J. Yu C. . (2025). Bone marrow mesenchymal stem cells-derived exosomal miR-24-3p alleviates spinal cord injury by targeting MAPK9 to inhibit the JNK/c-Jun/c-Fos pathway. Arch. Biochem. Biophys. 769:110434. doi: 10.1016/j.abb.2025.110434 40274174

[B43] Li D. Zhang P. Yao X. Li H. Shen H. Li X. . (2018). Exosomes derived from miR-133b-modified mesenchymal stem cells promote recovery after spinal cord injury. Front. Neurosci. 12:845. doi: 10.3389/fnins.2018.00845 30524227 PMC6262643

[B44] Li M. Zhang T. Li P. Luan Z. Liu J. Wang Y. . (2025). IL-4-primed human umbilical cord mesenchymal stem cells-derived extracellular vesicles facilitate recovery in spinal cord injury via the miR-21-5p/PDCD4-mediated shifting of macrophage M1/M2 polarization. Life Sci. 364:123441. doi: 10.1016/j.lfs.2025.123441 39909387

[B45] Li R. Y. Hu Q. Shi X. Luo Z. Y. Shao D. H. (2023). Crosstalk between exosomes and autophagy in spinal cord injury: fresh positive target for therapeutic application. Cell Tissue Res. 391, 1–17. doi: 10.1007/s00441-022-03699-6 36380098 PMC9839811

[B46] Li Y. Khan N. Ritzel R. M. Lei Z. Allen S. Faden A. I. . (2023). Sexually dimorphic extracellular vesicle responses after chronic spinal cord injury are associated with neuroinflammation and neurodegeneration in the aged brain. J. Neuroinflammation 20:197. doi: 10.1186/s12974-023-02881-z 37653491 PMC10469550

[B47] Li Y. Luo W. Meng C. Shi K. Gu R. Cui S. (2024). Exosomes as promising bioactive materials in the treatment of spinal cord injury. Stem Cell Res. Ther. 15:335. doi: 10.1186/s13287-024-03952-5 39334506 PMC11438208

[B48] Liang Z. Yang Z. Xie H. Rao J. Xu X. Lin Y. . (2024). Small extracellular vesicles from hypoxia-preconditioned bone marrow mesenchymal stem cells attenuate spinal cord injury via miR-146a-5p-mediated regulation of macrophage polarization. Neural Regen. Res. 19, 2259–2569. doi: 10.4103/1673-5374.391194 38488560 PMC11034578

[B49] Liao Z. Zeng J. Lin A. Zou Y. Zhou Z. (2025). Pre-treated mesenchymal stem cell-derived exosomes: a new perspective for accelerating spinal cord injury repair. Eur. J. Pharmacol. 992:177349. doi: 10.1016/j.ejphar.2025.177349 39921061

[B50] Lin M. Alimerzaloo F. Wang X. Alhalabi O. Krieg S. M. Skutella T. . (2025). Harnessing stem cell-derived exosomes: a promising cell-free approach for spinal cord injury. Stem Cell Res. Ther. 16:182. doi: 10.1186/s13287-025-04296-4 40247394 PMC12004558

[B51] Liu B. Zheng W. Dai L. Fu S. Shi E. (2022). Bone marrow mesenchymal stem cell derived exosomal miR-455-5p protects against spinal cord ischemia reperfusion injury. Tissue Cell. 74:101678. doi: 10.1016/j.tice.2021.101678 34823099

[B52] Liu J. Lin M. Qiao F. Zhang C. (2022). Exosomes derived from lncRNA TCTN2-modified mesenchymal stem cells improve spinal cord injury by miR-329-3p/IGF1R axis. J. Mol. Neurosci. 72, 482–495. doi: 10.1007/s12031-021-01914-7 34623606

[B53] Liu S. Jia J. Zhou H. Zhang C. Liu L. Liu J. . (2019). PTEN modulates neurites outgrowth and neuron apoptosis involving the PI3K/Akt/mTOR signaling pathway. Mol. Med. Rep. 20, 4059–4066. doi: 10.3892/mmr.2019.10670 31702028 PMC6797942

[B54] Liu W. Rong Y. Wang J. Zhou Z. Ge X. Ji C. . (2020). Exosome-shuttled miR-216a-5p from hypoxic preconditioned mesenchymal stem cells repair traumatic spinal cord injury by shifting microglial M1/M2 polarization. J. Neuroinflammation 17:47. doi: 10.1186/s12974-020-1726-7 32019561 PMC7001326

[B55] Liu X. Zhang Y. Wang Y. Qian T. (2021). Inflammatory response to spinal cord injury and its treatment. World Neurosurg. 155, 19–31. doi: 10.1016/j.wneu.2021.07.148 34375779

[B56] Luan X. Sansanaphongpricha K. Myers I. Chen H. Yuan H. Sun D. (2017). Engineering exosomes as refined biological nanoplatforms for drug delivery. Acta Pharmacol. Sin. 38, 754–763. doi: 10.1038/aps.2017.12 28392567 PMC5520184

[B57] Ma K. Xu H. Zhang J. Zhao F. Liang H. Sun H. . (2019). Insulin-like growth factor-1 enhances neuroprotective effects of neural stem cell exosomes after spinal cord injury via an miR-219a-2-3p/YY1 mechanism. Aging 11, 12278–12294. doi: 10.18632/aging.102568 31848325 PMC6949049

[B58] Ma L. Yang Y. Chen T. Ma L. Deng Q. (2025). Developing an NT3-loaded exosomal biodegradable conductive hydrogel combined with EA for targeted treatment of spinal cord injury. Mater. Today Bio. 33:101988. doi: 10.1016/j.mtbio.2025.101988 40605987 PMC12213280

[B59] Ma Y. Yu X. Pan J. Wang Y. Li R. Wang X. . (2025). Exosomes: a promising microenvironment modulator for spinal cord injury treatment. Int. J. Biol. Sci. 21, 3791–824. doi: 10.7150/ijbs.115242 40520019 PMC12160932

[B60] Milich L. M. Ryan C. B. Lee J. K. (2019). The origin, fate, and contribution of macrophages to spinal cord injury pathology. Acta Neuropathol. 137, 785–797. doi: 10.1007/s00401-019-01992-3 30929040 PMC6510275

[B61] Nakagawa H. Ninomiya T. Yamashita T. Takada M. (2019). Treatment with the neutralizing antibody against repulsive guidance molecule-a promotes recovery from impaired manual dexterity in a primate model of spinal cord injury. Cereb. Cortex 29, 561–572. doi: 10.1093/cercor/bhx338 29315368

[B62] Nakazaki M. Morita T. Lankford K. L. Askenase P. W. Kocsis J. D. (2021). Small extracellular vesicles released by infused mesenchymal stromal cells target M2 macrophages and promote TGF-β upregulation, microvascular stabilization and functional recovery in a rodent model of severe spinal cord injury. J. Extracell. Vesicles 10:e12137. doi: 10.1002/jev2.12137 34478241 PMC8408371

[B63] Nie H. Jiang Z. (2021). Bone mesenchymal stem cell-derived extracellular vesicles deliver microRNA-23b to alleviate spinal cord injury by targeting toll-like receptor TLR4 and inhibiting NF-κB pathway activation. Bioengineered 12, 8157–8172. doi: 10.1080/21655979.2021.1977562 34663169 PMC8806461

[B64] Noble L. J. Wrathall J. R. (1989). Distribution and time course of protein extravasation in the rat spinal cord after contusive injury. Brain Res. 482, 57–66. doi: 10.1016/0006-8993(89)90542-8 2706482

[B65] Okada S. (2016). The pathophysiological role of acute inflammation after spinal cord injury. Inflammation Regen. 36:20. doi: 10.1186/s41232-016-0026-1 29259693 PMC5725917

[B66] Orr M. B. Gensel J. C. (2018). Spinal cord injury scarring and inflammation: therapies targeting glial and inflammatory responses. Neurotherapeutics 15, 541–553. doi: 10.1007/s13311-018-0631-6 29717413 PMC6095779

[B67] Ortega M. A. Fraile-Martinez O. García-Montero C. Haro S. Álvarez-Mon M. Á. De Leon-Oliva D. . (2023). A comprehensive look at the psychoneuroimmunoendocrinology of spinal cord injury and its progression: mechanisms and clinical opportunities. Mil. Med. Res. 10:26. doi: 10.1186/s40779-023-00461-z 37291666 PMC10251601

[B68] Pan D. Liu W. Zhu S. Fan B. Yu N. Ning G. . (2021). Potential of different cells-derived exosomal microRNA cargos for treating spinal cord injury. J. Orthop. Transl. 31, 33–40. doi: 10.1016/j.jot.2021.09.008 34760623 PMC8560648

[B69] Picco F. Zeboudj L. Oggero S. Prato V. Burgoyne T. Gamper N. . (2025). Macrophage to neuron communication via extracellular vesicles in neuropathic pain conditions. Heliyon 11:e41268. doi: 10.1016/j.heliyon.2024.e41268 39811367 PMC11730208

[B70] Qin T. Li C. Xu Y. Qin Y. Jin Y. He R. . (2024). Local delivery of EGFR(+)NSCs-derived exosomes promotes neural regeneration post spinal cord injury via miR-34a-5p/HDAC6 pathway. Bioactive Mater. 33, 424–443. doi: 10.1016/j.bioactmat.2023.11.013 38059122 PMC10696309

[B71] Ralph P. C. Choi S. W. Baek M. J. Lee S. J. (2024). Regenerative medicine approaches for the treatment of spinal cord injuries: progress and challenges. Acta Biomater. 189, 57–72. doi: 10.1016/j.actbio.2024.10.021 39424019

[B72] Ransohoff R. M. (2016). How neuroinflammation contributes to neurodegeneration. Sci. 353, 777–783. doi: 10.1126/science.aag2590 27540165

[B73] Shang J. Xu H. Xie L. Lv H. Wang F. Jin C. . (2025). Global trends on exosomes in spinal cord injury: a bibliometric analysis and mini-review. Biomater. Transl. 6, 151–164. doi: 10.12336/bmt.24.00004 40641991 PMC12237805

[B74] Shao M. Ye S. Chen Y. Yu C. Zhu W. (2024). Exosomes from hypoxic ADSCs ameliorate neuronal damage post spinal cord injury through circ-Wdfy3 delivery and inhibition of ferroptosis. Neurochem. Int. 177:105759. doi: 10.1016/j.neuint.2024.105759 38735393

[B75] Silva N. A. Sousa N. Reis R. L. Salgado A. J. (2014). From basics to clinical: a comprehensive review on spinal cord injury. Prog. Neurobiol. 114, 25–57. doi: 10.1016/j.pneurobio.2013.11.002 24269804

[B76] Silvestro S. Mazzon E. (2022). MiRNAs as promising translational strategies for neuronal repair and regeneration in spinal cord injury. Cells 11:2177. doi: 10.3390/cells11142177 35883621 PMC9318426

[B77] Singh N. Guha L. Kumar H. (2024). From hope to healing: exploring the therapeutic potential of exosomes in spinal cord injury. Extracell. Vesicle 3:100044. doi: 10.1016/j.vesic.2024.100044

[B78] Sousa C. S. Monteiro A. Salgado A. J. Silva N. A. (2025). Combinatorial therapies for spinal cord injury repair. Neural Regen. Res. 20, 1293–1308. doi: 10.4103/NRR.NRR-D-24-00061 38845223 PMC11624878

[B79] Sugai K. Nakamura M. Okano H. Nagoshi N. (2025). Stem cell therapies for spinal cord injury in humans: a review of recent clinical research. Brain Spine 5:104207. doi: 10.1016/j.bas.2025.104207 40027291 PMC11870206

[B80] Sun Y. Liu Q. Qin Y. Xu Y. Zhao J. Xie Y. . (2024a). Exosomes derived from CD271+CD56+ bone marrow mesenchymal stem cell subpopoulation identified by single-cell RNA sequencing promote axon regeneration after spinal cord injury. Theranostics 14, 510–527. doi: 10.7150/thno.89008 38169566 PMC10758065

[B81] Sun Y. Zhao J. Liu Q. Xu Y. Qin Y. He R. . (2024b). Intranasal delivery of small extracellular vesicles from specific subpopulation of mesenchymal stem cells mitigates traumatic spinal cord injury. J. Control Release. 369, 335–350. doi: 10.1016/j.jconrel.2024.03.037 38519036

[B82] Tan F. Li X. Wang Z. Li J. Shahzad K. Zheng J. (2024). Clinical applications of stem cell-derived exosomes. Sig. Transduct Target Ther. 9:17. doi: 10.1038/s41392-023-01704-0 38212307 PMC10784577

[B83] Umezu T. Ohyashiki K. Kuroda M. Ohyashiki J. H. (2013). Leukemia cell to endothelial cell communication via exosomal miRNAs. Oncogene 32, 2747–2755. doi: 10.1038/onc.2012.295 22797057

[B84] Visavadiya N. P. Patel S. P. VanRooyen J. L. Sullivan P. G. Rabchevsky A. G. (2016). Cellular and subcellular oxidative stress parameters following severe spinal cord injury. Redox Biol. 8, 59–67. doi: 10.1016/j.redox.2015.12.011 26760911 PMC4712315

[B85] Wang G. Shi Y. Jiang X. Leak R. K. Hu X. Wu Y. . (2015). HDAC inhibition prevents white matter injury by modulating microglia/macrophage polarization through the GSK3β/PTEN/Akt axis. Proc. Natl. Acad. Sci. U.S.A. 112, 2853–2858. doi: 10.1073/pnas.1501441112 25691750 PMC4352818

[B86] Wang J. Chen S. Sawant H. Chen Y. Bihl J. C. (2024). The miR-210 primed endothelial progenitor cell exosomes alleviate acute ischemic brain injury. Curr. Stem Cell Res. Ther. 19, 1164–1174. doi: 10.2174/011574888X266357230923113642 37957914 PMC11082070

[B87] Wang Y. Lai X. Wu D. Liu B. Wang N. Rong L. (2021). Umbilical mesenchymal stem cell-derived exosomes facilitate spinal cord functional recovery through the miR-199a-3p/145-5p-mediated NGF/TrkA signaling pathway in rats. Stem Cell Res. Ther. 12:117. doi: 10.1186/s13287-021-02148-5 33579361 PMC7879635

[B88] Wang Z. Bi H. Li D. Zhang W. Wang C. Yang J. . (2025). Bibliometric analysis of exosome research in spinal cord injury (2000–May 2024). Trends, collaborations, and emerging insights. Drug Des. Dev. Ther. 19, 6829–6848. doi: 10.2147/DDDT.S522129 40799317 PMC12341566

[B89] Wei Z. Guo C. Zhou H. Wu Y. Zhou X. Chen J. . (2025). Exosome-mediated miRNA delivery: a molecular switch for reshaping neuropathic pain therapy. Front. Mol. Neurosci. 18:1625943. doi: 10.3389/fnmol.2025.1625943 40688695 PMC12271170

[B90] Wu J. Li X. Wang Q. Wang S. He W. Wu Q. . (2022). LncRNA/miRNA/mRNA ceRNA network analysis in spinal cord injury rat with physical exercise therapy. PeerJ 10:e13783. doi: 10.7717/peerj.13783 35923891 PMC9341448

[B91] Wu Y. Wang Y. Zhou J. Tang Z. Huang L. Liu S. (2025). How advanced are exosomes as cell-free therapeutics for spinal cord injury?. Int. J. Nanomed. 20, 11669–11683. doi: 10.2147/IJN.S536652 41019233 PMC12466603

[B92] Xiao C. L. Yin W. C. Zhong Y. C. Luo J. Q. Liu L. L. Liu W. Y. . (2022). The role of PI3K/Akt signalling pathway in spinal cord injury. Biomed. Pharmacother. 156:113881. doi: 10.1016/j.biopha.2022.113881 36272264

[B93] Xiao X. Li W. Rong D. Xu Z. Zhang Z. Ye H. . (2021). Human umbilical cord mesenchymal stem cells-derived extracellular vesicles facilitate the repair of spinal cord injury via the miR-29b-3p/PTEN/Akt/mTOR axis. Cell Death Discov. 7:212. doi: 10.1038/s41420-021-00572-3 34381025 PMC8357833

[B94] Xin H. Li Y. Cui Y. Yang J. J. Zhang Z. G. Chopp M. (2013). Systemic administration of exosomes released from mesenchymal stromal cells promote functional recovery and neurovascular plasticity after stroke in rats. J. Cereb. Blood Flow Metab. 33, 1711–1715. doi: 10.1038/jcbfm.2013.152 23963371 PMC3824189

[B95] Xu G. Ao R. Zhi Z. Jia J. Yu B. (2019a). miR-21 and miR-19b delivered by hMSC-derived EVs regulate the apoptosis and differentiation of neurons in patients with spinal cord injury. J. Cell Physiol. 234, 10205–10217. doi: 10.1002/jcp.27690 30387159

[B96] Xu L. Cao H. Xie Y. Zhang Y. Du M. Xu X. . (2019b). Exosome-shuttled miR-92b-3p from ischemic preconditioned astrocytes protects neurons against oxygen and glucose deprivation. Brain Res. 1717, 66–73. doi: 10.1016/j.brainres.2019.04.009 30986407

[B97] Yang Z. Liang Z. Rao J. Xie H. Zhou M. Xu X. . (2024). Hypoxic-preconditioned mesenchymal stem cell-derived small extracellular vesicles promote the recovery of spinal cord injury by affecting the phenotype of astrocytes through the miR-21/JAK2/STAT3 pathway. CNS Neurosci. Ther. 30:e14428. doi: 10.1111/cns.14428 37641874 PMC10915983

[B98] Yang Z. L. Rao J. Lin F. B. Liang Z. Y. Xu X. J. Lin Y. K. . (2022). The role of exosomes and exosomal noncoding RNAs from different cell sources in spinal cord injury. Front. Cell Neurosci. 16:882306. doi: 10.3389/fncel.2022.882306 35518647 PMC9062236

[B99] Yi D. Zhang Y. Li M. Chen J. Chen X. Wang L. . (2023). Ultrasound-targeted microbubble destruction assisted delivery of platelet-rich plasma-derived exosomes promoting peripheral nerve regeneration. Tissue Eng. Part A 29, 645–662. doi: 10.1089/ten.tea.2023.0133 37612613

[B100] Yu T. Yang L. L. Zhou Y. Wu M. F. Jiao J. H. (2024). Exosome-mediated repair of spinal cord injury: a promising therapeutic strategy. Stem Cell Res. Ther. 15:6. doi: 10.1186/s13287-023-03614-y 38167108 PMC10763489

[B101] Yu T. Zhao C. Hou S. Zhou W. Wang B. Chen Y. (2019). Exosomes secreted from miRNA-29b-modified mesenchymal stem cells repaired spinal cord injury in rats. Braz. J. Med. Biol. Res. 52:e8735. doi: 10.1590/1414-431X20198735 31826179 PMC6903804

[B102] Yuan F. Peng W. Yang Y. Xu J. Liu Y. Xie Y. . (2023). Endothelial progenitor cell-derived exosomes promote anti-inflammatory macrophages via SOCS3/JAK2/STAT3 axis and improve the outcome of spinal cord injury. J. Neuroinflammation 20:156. doi: 10.1186/s12974-023-02833-7 37391774 PMC10314438

[B103] Zha X. (2025). Exosome-based therapy for spinal cord injury: a narrative review. Adv. Techno. Neurosci. 2, 128–134. doi: 10.4103/ATN.ATN-D-25-00001

[B104] Zhang A. Bai Z. Yi W. Hu Z. Hao J. (2021). Overexpression of miR-338-5p in exosomes derived from mesenchymal stromal cells provides neuroprotective effects by the Cnr1/Rap1/Akt pathway after spinal cord injury in rats. Neurosci. Lett. 761:136124. doi: 10.1016/j.neulet.2021.136124 34302891

[B105] Zhang C. Talifu Z. Xu X. Liu W. Ke H. Pan Y. . (2023). MicroRNAs in spinal cord injury: a narrative review. Front. Mol. Neurosci. 16:1099256. doi: 10.3389/fnmol.2023.1099256 36818651 PMC9931912

[B106] Zhang L. Han P. (2022b). Neural stem cell-derived exosomes suppress neuronal cell apoptosis by activating autophagy via miR-374-5p/STK-4 axis in spinal cord injury. J. Musculoskelet. Neuronal Interact. 22, 411–421. 36046998 PMC9438516

[B107] Zhang Y. Chopp M. Meng Y. Katakowski M. Xin H. Mahmood A. . (2015). Effect of exosomes derived from multipluripotent mesenchymal stromal cells on functional recovery and neurovascular plasticity in rats after traumatic brain injury. J. Neurosurg. 122, 856–867. doi: 10.3171/2014.11.JNS14770 25594326 PMC4382456

[B108] Zhang Y. Wang J. Yang B. Qiao R. Li A. Guo H. . (2020). Transfer of MicroRNA-216a-5p from exosomes secreted by human urine-derived stem cells reduces renal ischemia/reperfusion injury. Front. Cell Dev. Biol. 8:610587. doi: 10.3389/fcell.2020.610587 33415108 PMC7783217

[B109] Zrzavy T. Schwaiger C. Wimmer I. Berger T. Bauer J. Butovsky O. . (2021). Acute and non-resolving inflammation associate with oxidative injury after human spinal cord injury. Brain 144, 144–161. doi: 10.1093/brain/awaa360 33578421 PMC7880675

